# Initially Normal MRI, Delayed Splenial Lesion in Marchiafava‐Bignami Disease

**DOI:** 10.1002/ccr3.71926

**Published:** 2026-01-22

**Authors:** Tsuyoshi Nojima, Ryo Tanabe, Takafumi Obara, Takashi Hongo, Tetsuya Yumoto

**Affiliations:** ^1^ Department of Emergency, Critical Care, and Disaster Medicine, Faculty of Medicine Dentistry and Pharmaceutical Sciences, Okayama University Okayama Okayama Prefecture Japan; ^2^ Ohta Hospital Niimi Okayama Prefecture Japan

**Keywords:** alcohol, Marchiafava–Bignami disease, MRI, thiamine

## Abstract

Marchiafava–Bignami disease (MBD) may show normal findings on early MRI. In patients with alcohol use disorder or risk of thiamine deficiency, repeat imaging is important because splenial lesions can develop later. Recognizing this pattern is key to avoiding delayed diagnosis.

Marchiafava–Bignami disease (MBD) is classically associated with chronic alcohol use and thiamine deficiency, although cases without these factors have also been reported [1]. The disease is characterized by demyelination of the corpus callosum, most commonly affecting the splenium, as seen on magnetic resonance imaging (MRI) [1]. We describe a patient who had initially normal diffusion‐weighted imaging (DWI) but later developed a splenial lesion, highlighting the diagnostic challenge of early MBD.

A 73‐year‐old man with chronic alcohol use was admitted to a local hospital for mild right hemiparesis, without aphasia or other neurologic deficits. On admission, his vital signs were stable. Brain MRI performed 3 h after symptom onset showed no abnormalities on DWI. Magnetic resonance angiography (MRA) demonstrated patency of the major intracranial arteries. However, fluid‐attenuated inversion recovery (FLAIR) revealed a faint hyperintensity in the splenium of the corpus callosum (Figure 1A). He was hospitalized overnight without vitamin supplementation and discharged after his symptoms resolved.

**FIGURE 1:**
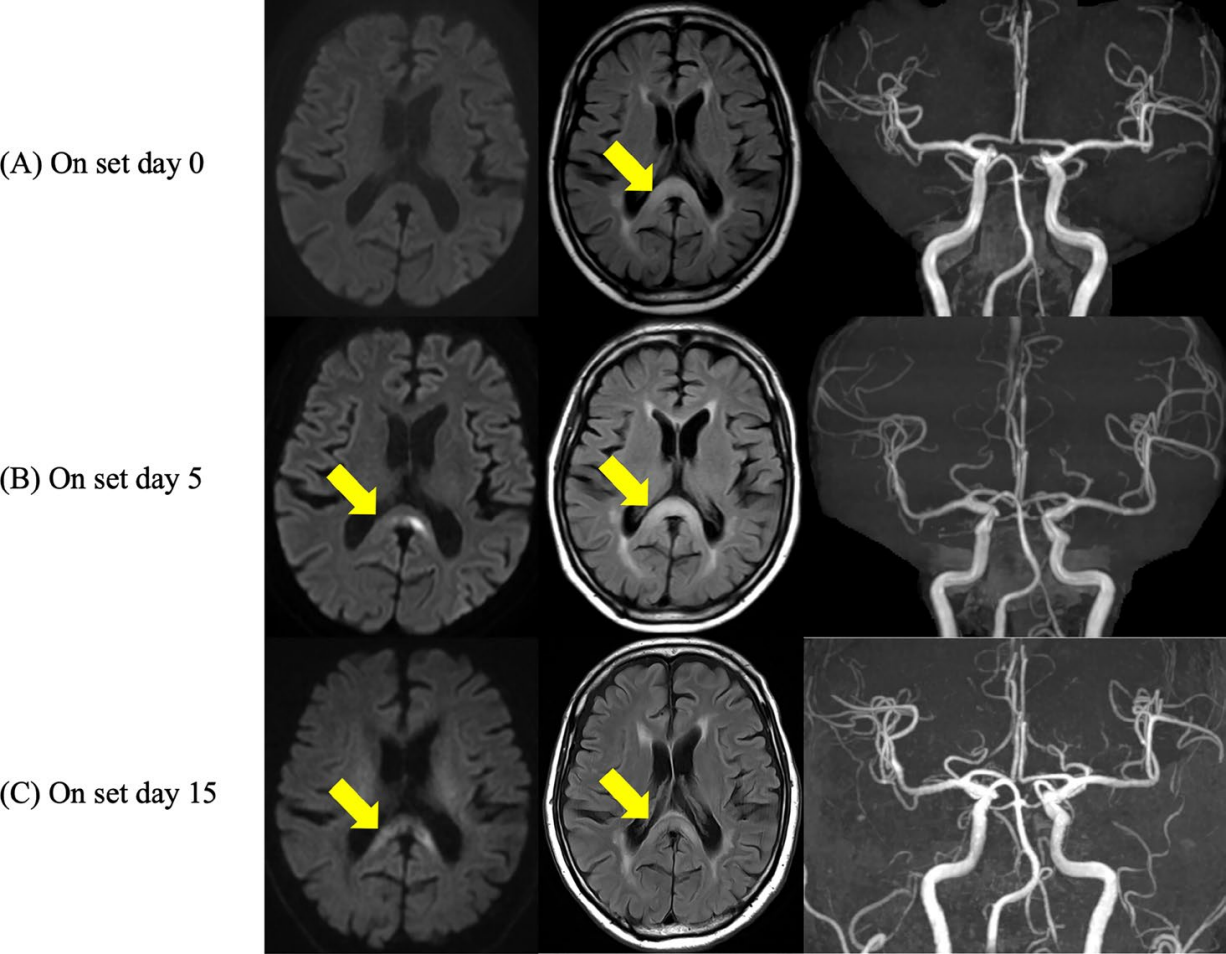
Brain MRI in this case. (A) Day 0: faint splenial hyperintensity. (B) Day 5: marked hyperintensity on FLAIR/DWI (arrows). (C) Day 15: decreased signal after thiamine (arrows).

Five days later, the right hemiparesis recurred, and he was transferred to our institution. Repeat MRI showed marked hyperintensity in the splenium on FLAIR and DWI (Figure 1B). MRA again confirmed preserved intracranial circulation. Laboratory tests revealed normal glucose and electrolyte levels, with no evidence of seizure activity, but the thiamine level was markedly reduced (14 ng/mL; normal > 24). Intravenous thiamine (500 mg/day) was initiated, leading to gradual improvement and complete recovery by hospital day 4. On hospital day 9 (day 15 after onset), follow‐up MRI showed decreased splenial signal intensity on FLAIR and DWI, consistent with radiologic improvement after thiamine therapy (Figure 1C). He was discharged on hospital day 15 (day 20 after onset) with sustained recovery, although he declined long‐term follow‐up.

This case highlights the risk of delayed diagnosis in MBD. The patient presented with transient hemiparesis and an initially normal DWI result, but a splenial lesion appeared on follow‐up imaging. MBD should remain in the differential diagnosis of transient neurologic symptoms in patients with alcohol use disorder, even when early MRI findings are unremarkable. Recognizing this temporal evolution is crucial to avoid misdiagnosis as stroke and to ensure timely thiamine supplementation.

MBD occurs in the setting of chronic alcohol use and thiamine deficiency [1, 2], predisposing the corpus callosum to metabolic and structural injury [1, 3]. The hallmark finding is splenial hyperintensity on DWI [2, 3], but early abnormalities may be limited to subtle changes of FLAIR, with DWI positivity appearing later [2, 3]. This temporal evolution likely reflects delayed intramyelinic edema caused by thiamine depletion [2]. Although splenial lesions are well documented in MBD, few reports have described initially normal DWI followed by delayed abnormalities [2], as observed in our patient. This patient presented with right hemiparesis; however, the lateral predominance of such symptoms remains unclear in the literature.

MBD may present with transient neurologic deficits and an initially normal DWI result, with splenial lesions becoming evident only on follow‐up imaging. Clinicians should recognize that MRI abnormalities may appear later and that repeat imaging is essential if symptoms recur or persist.

## Author Contributions

**Tsuyoshi Nojima:** conceptualization, investigation, project administration, visualization, writing – original draft. **Ryo Tanabe:** resources, writing – review and editing. **Takafumi Obara:** writing – review and editing. **Takashi Hongo:** writing – review and editing. **Tetsuya Yumoto:** supervision, writing – review and editing.

## Funding

The authors have nothing to report.

## Consent

Written informed consent was obtained from the patient for the publication of this case report including the images.

## Conflicts of Interest

The authors declare no conflicts of interest.

## Data Availability

The authors have nothing to report.
